# Palpebral myiasis due *Cochliomyia macellaria* in an alcoholic patient

**DOI:** 10.1590/0037-8682-0168-2020

**Published:** 2020-11-13

**Authors:** Luis Arthur Brasil Gadelha Farias, Maria Jânia Teixeira, Roberto da Justa Pires

**Affiliations:** 1 Universidade Federal do Ceará, Faculdade de Medicina, Fortaleza, CE, Brasil.; 2 Secretaria de Saúde do Estado do Ceará, Hospital São José de Doenças Infecciosas, Fortaleza, CE, Brasil.; 3 Universidade Federal do Ceará, Departamento de Parasitologia Médica, Fortaleza, CE, Brasil.

**FIGURE 1:**
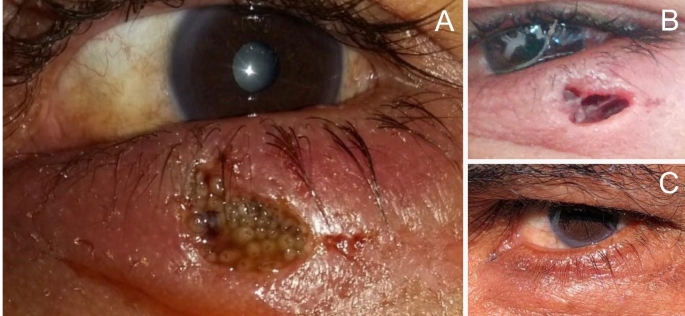
Palpebral myiasis. **(A)**: Ulcerated lesion of the eyelid, with erythematous and swollen edges, presenting numerous larvae of *Cochliomyia macellaria*; **(B):** Aspect of the lesion after manual removal of larvae and local cleansing. **(C):** Cicatricial aspect of the lesion after seven days of treatment.

A 51-year-old man with a history of alcoholism, with no associated comorbidities, and who suffered a trauma after excessive alcohol consumption two weeks ago, presented to the emergency room with a 2-week history of erythema, pain, and a “moving worms” sensation in the inferior eyelid of his left eye. Physical examination revealed an ulcerated lesion with erythematous and swollen edges containing numerous larvae (Figure 1A). He was treated with a single dose of ivermectin (6 mg/day), and the larvae were mechanically removed (Figure 1B). Antibiotics were not necessary. The larvae were identified as *Cochliomyia macellaria*, a common causative agent of myiasis in northeastern Brazil. These flies can be found in the Americas, mostly in the neotropical region, but are also seen in areas spanning the Arctic region to southern Canada. Treatment of this infection in humans involves the mechanical removal of the larvae and the administration of anti-parasitic drugs^1^. Our patient was discharged after four days upon complete resolution of the lesion. At the 2-month follow-up, he did not report any recurrence (Figure 1C).

Human myiasis cases are commonly related to risk factors such as poor hygiene, alcoholism, trauma, senility, mental or neurological diseases, immunosuppression, diabetes, malnutrition, and suppurating lesions. We hypothesized that trauma was the most probable cause for the development of myiasis in an alcoholic and abandoned person. Similar cases of alcoholism-related myiasis have been reported involving the oral cavity and the ear^2^ ^,^ ^3^. Myiasis is still a neglected tropical disease that requires greater medical understanding and prompt recognition.
